# Supplementary material for: Label-free neuroimaging in vivo using synchronous angular scanning microscopy with single-scattering accumulation algorithm
Source: Nat Commun. 2019 Jul 17;10:3152. doi: 10.1038/s41467-019-11040-z (PMC6637127; doi:10.1038/s41467-019-11040-z)
Supplement: Supplementary file 1 — Supplementary Information [file 41467_2019_11040_MOESM1_ESM.pdf]

Supplementary Information

Label-free neuroimaging in vivo using synchronous angular scanning microscopy with single-scattering accumulation algorithm

Kim *et al.*

Label-free neuroimaging in vivo using synchronous angular scanning microscopy with single-scattering accumulation algorithm

Moonseok Kim[1,2,3,4,+], Yonghyeon Jo[1,2,+], Jin Hee Hong[1,2], Suhyun Kim[5], Seokchan Yoon[1,2],

Kyung-Deok Song[1,2], Sungsam Kang[6], Byunghak Lee[7], Guang Hoon Kim[7],

Hae-Chul Park[5], and Wonshik Choi[1,2,*]

[1]*Center for Molecular Spectroscopy and Dynamics, Institute for Basic Science, Seoul 02841, Korea*
[2]*Department of Physics, Korea University, Seoul 02841, Korea*
[3]*Department of Medical Life Sciences, College of Medicine, The Catholic University of Korea, Seoul, 06591, Korea*
[4]*Department of Biomedicine & Health Sciences, College of Medicine, The Catholic University of Korea, Seoul, 06591 Korea*
[5]*Department of Biomedical Sciences, Korea University, Ansan 425-707, Korea*
[6]*Laser Biomedical Research Center, G. R. Harrison Spectroscopy Laboratory, Massachusetts Institute of Technology, Cambridge, Massachusetts 02139, USA*
[7]*Korea Electrotechnology Research Institute, Ansan 15588, Korea*
[+]*Moonseok Kim and Yonghyeon Jo contributed equally to this work.*
[*]*wonshik@korea.ac.kr*

Supplementary Note 1: Detailed experimental setup

Detailed layout of the experimental setup is shown in Supplementary Fig. 1. For the experimental demonstration of AO-SASM, we used a broad-band supercontinuum laser (Laser 1, NKT Photonics, model EXR-15). The center wavelength and bandwidth of Laser 1 were tunable. The output beam from the laser was sent to a galvanometer scanning mirror (GM, Cambridge Technology 6220H) for scanning the angle of illumination, and then divided into sample beam and reference beam at a polarizing beam splitter (PBS). The sample beam reflected off from the PBS was sent to the objective lens (OL, 40X Nikon CFI Plan Fluorite Objective 0.8 NA 2 mm WD) to illuminate the sample. The backscattered wave from the sample was captured by the OL and sent to the camera (PCO. edge rolling shutter 4.2). The sample beam was magnified by a factor of 120 via the relay optics to the camera plane. The reference beam transmitted through the PBS was reflected from the reference mirror (RM), which was mounted on the translation stage to adjust optical path length for temporal gating of interferometric detection. It was then sent to the diffraction grating (DG, Edmund Optics, 120 lp/mm), and its first order diffraction was selected by the iris diaphragm (ID) and relayed to the camera to form an interferogram with the sample beam. A wave plate (WP) was used to set the

polarization of the reference beam to be the same as that of the sample beam. Confocal fluorescence/reflectance microscopes were superposed on the top of AO-SASM. For confocal fluorescence imaging, a DPSS laser with the wavelength of 473 nm (Laser 2, LSR473NL-300) was installed for the excitation of green fluorescent proteins. The flip mirrors 1, 2, 3 and flip BS were set up to scan a focused beam and de-scan the fluorescence signal by GM. Then, pinhole-gated signal was reflected by the dichroic mirror and detected by the PMT 2. For confocal reflectance imaging, the output from the supercontinuum laser was used as a light source by turning off flip mirror 3, and PMT 1 was used for detecting the pinhole-gated elastic backscattering signal.

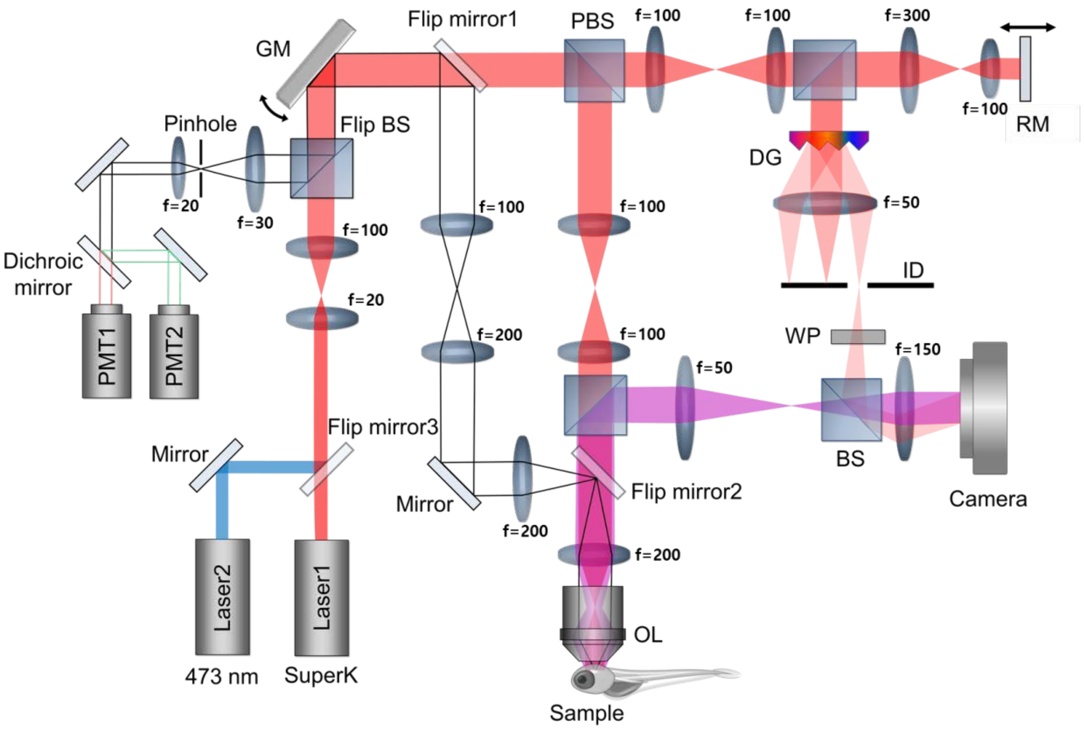

Supplementary Figure 1. Detailed experimental setup. Laser 1: broad-band supercontinuum laser, NKT Photonics, model EXR-15, GM: a galvanometer scanning mirror, PBS: a polarizing beam splitter, OL: the objective lens, RM: the reference mirror, DG: the diffraction grating, ID: the iris diaphragm, WP: A wave plate, Laser 2: a DPSS laser with the wavelength of 473 nm.

Supplementary Note 2: The rationale of synchronous angular scanning for the fast recording of a time-gated reflection matrix

The use of a short-coherence-length light source for the wide-field interferometric imaging needs a special care especially when the angle of sample beam is rotated by a scanning mirror. If the pulse front of the reference wave is fixed and parallel to the camera plane, the interference between sample and reference waves occur only within a narrow range set by the coherence length of light source. To clarify, let us consider that the pulse front of the sample wave indicated as a red line in Supplementary Fig. 2a has angle θ_{d} with respect to the pulse front of the reference wave (black line) at the camera plane. With the increase of the distance x from the center of the camera, the path length difference ΔL between sample and reference waves increases as $\Delta L = x \times tan\theta_{\mathrm{d}}$. Since the interference between sample and reference waves can occur only when $\Delta L \leq l_{\mathrm{c}}$, where l_{c} is the coherence length of the light source, the width of interference is given by $\Delta x_{\mathrm{d}} = 2l_{\mathrm{c}}/tan\theta_{\mathrm{d}}$ at the camera plane. For the case when the magnification from sample plane to the camera plane is M, we can obtain the width of interference Δx_{s} at the sample plane by using the relations, $\Delta x_{\mathrm{d}} = M \times \Delta x_{\mathrm{s}}$ and $tan\theta_{\mathrm{d}} \approx sin\theta_{\mathrm{d}} = sin\theta_{\mathrm{s}}/M$. Here θ_{s} is the angle of illumination of the sample wave at the sample plane. In the case of maximum illumination angle where $sin\theta_{\mathrm{m}} = NA$, the width of interference at the sample plane is given by $\Delta x_{\mathrm{s}} = 2l_{\mathrm{c}}/NA$. In our experimental condition where $l_{\mathrm{c}} = 15\ \mu\mathrm{m}$ and $NA = 0.8$, Δx_{s} is about $37.5\ \mu\mathrm{m}$. This is much smaller than the typical width of the field of view, which was $110\ \mu\mathrm{m}$. Supplementary Figures 2b and c show images for the case of a fixed reference. Interference occurred only in the narrow region for the case of the maximum oblique illumination, i.e. $sin\theta_{\mathrm{s}} = 0.8$ (Supplementary Fig. 2c), while interference occurred in entire field of view at normal illumination (Supplementary Fig. 2b). Measured FWHM of interference image in Supplementary Fig. 2c was $35\ \mu\mathrm{m}$, which is in good agreement with the predicted value. Inset in Supplementary Fig. 2c is magnified image of the dashed white box and shows a severe phase ramp due to the difference in angle between sample and reference waves. On the contrary, in case of rotating reference, interference was uniform over the entire view field for both normal (Supplementary Fig. 2d) and the maximum oblique angle (Supplementary Fig. 2e) illuminations.

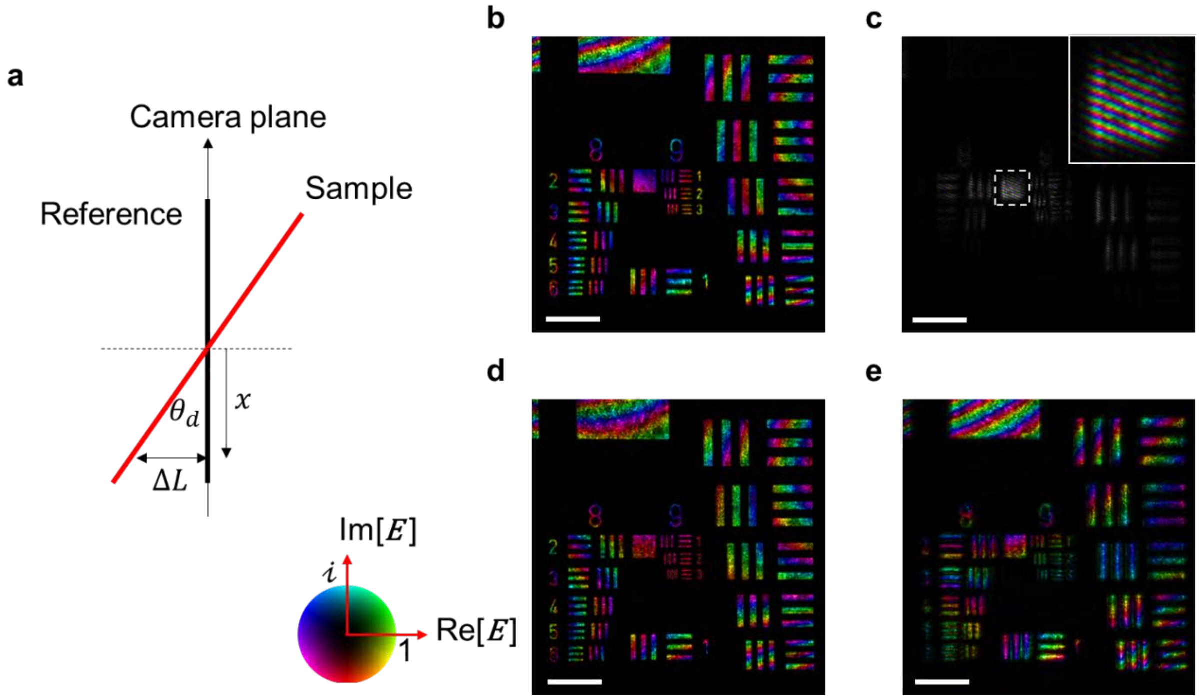

Supplementary Figure 2. Comparison between the fixed and rotating reference waves in the recording of time-gated complex-field maps. a, The pulse front of the sample wave indicated as a red line, the pulse front of the reference wave indicated as a black line. **b, c,** Complex field maps acquired for the case of a fixed reference with the normal illumination and with the maximum oblique illumination, respectively. **d, e,** Complex field maps acquired for the case of synchronous rotation of sample and reference waves with the normal illumination and with the maximum oblique illumination, respectively. Scale bar, 20 μm. The saturation and color of the color bar indicates the amplitude and phase of the complex field, respectively.

Volumetric imaging rate of our system depends on N_{in} and the camera frame rate. The depth sampling is equal to the coherence length of the source, which was 15 μm in our experiment, and axial resolution was about 2 μm. Therefore, there are at least 7 successive optical sections at axial resolution within a single coherence volume, from which more *en face* images can be retrieved along the axial direction by computational refocusing for better 3D visualization. If 100 angular images are used with 400 frames/s, then we could obtain images over the volume of 22×22×15 μm^3 in 0.25 seconds. If multiple scattering and aberration are more pronounced, then N_{in} should be increased to ensure enough SNR to resolve the target structures. Our camera frame rate was 60 fps for the view field of 110×110 μm^2, which corresponds to the volume image rate of 110×110×15 μm^3 per 1.67 seconds.

Supplementary Note 3: Coordinate transformation from the rotating reference frame to the laboratory frame

As described in the main text, AO-SASM measures the interferogram with respect to the rotating reference wave in the off-axis interferometric configuration, delicate procedure of image processing, including Hilbert transform and coordinate transform from the rotating reference frame to the laboratory frame, is required to extract the time-gated reflection matrix and the wavefront aberration as follows.

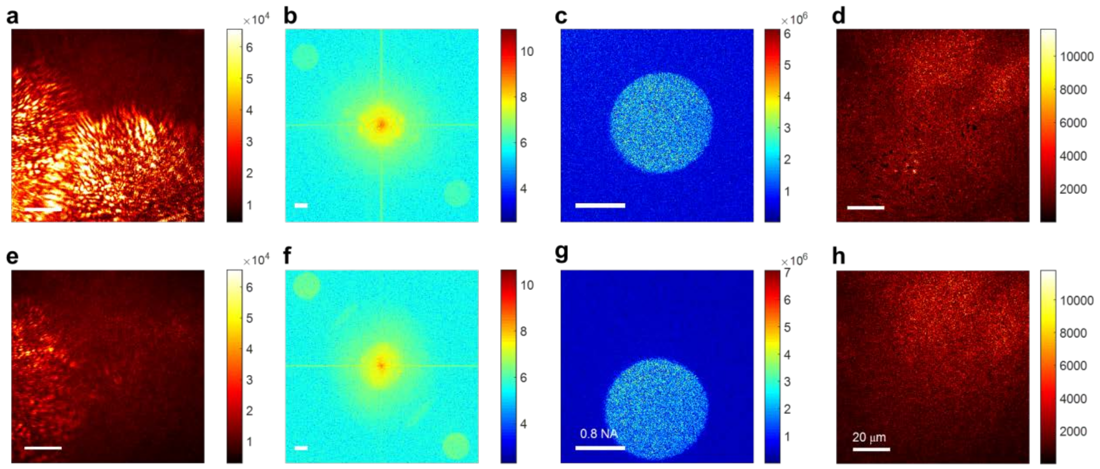

Supplementary Figure 3. Image processing in the off-axis interferometry. a, e, The interferograms of zebrafish for normal incidence and k^i/k_0=0.8, respectively. **b, f** The Fourier transform of the interferograms of (a), and (e). **c, g,** The angular spectrums of complex field maps in rotating frame for (b), and (f). **d, h,** The complex field maps obtained by the inverse Fourier transform for (c), and (g). Scale bar, 20 μm (**a, d, e, h**), 0.8 k_0NA (**b, c, f, g**). Color bars, intensity in arbitrary unit.

In the camera, the measured interferogram in AO-SASM is given by $I(\mathbf{r}_\mathrm{o};\mathbf{k}^\mathrm{i},\tau_0) = |E_\mathrm{S}(\mathbf{r}_\mathrm{o};\mathbf{k}^\mathrm{i},\tau_0) + E_\mathrm{R}(\mathbf{r}_\mathrm{o})|^2 = E_\mathrm{S}E_\mathrm{S}{}^* + E_\mathrm{R}E_\mathrm{R}{}^* + E_\mathrm{S}(\mathbf{r}_\mathrm{o};\mathbf{k}^\mathrm{i},\tau_0)E_\mathrm{R}(\mathbf{r}_\mathrm{o})^* + E_\mathrm{S}(\mathbf{r}_\mathrm{o};\mathbf{k}^\mathrm{i},\tau_0)^* E_R(\mathbf{r}_\mathrm{o}) = I_\mathrm{S} + I_\mathrm{R} + E_\mathrm{S}E_\mathrm{R}{}^* + E_\mathrm{S}{}^*E_\mathrm{R}$. Supplementary Figures 3a and 3e show the interferograms of zebrafish for normal incidence and k^i/k_0=0.8, respectively. By taking the Fourier transform of $I(\mathbf{r}_\mathrm{o};\mathbf{k}^\mathrm{i},\tau_0)$, angular spectrum $\tilde{I}(\mathbf{k};\mathbf{k}^\mathrm{i},\tau_0) = \tilde{I}_\mathrm{S}(\mathbf{k}) + \tilde{I}_\mathrm{R}(\mathbf{k}) + \widetilde{E_\mathrm{S}E_\mathrm{R}{}^*}(\mathbf{k}-\mathbf{k}^\mathrm{i}-\mathbf{k}_\mathrm{DG}) + \widetilde{E_\mathrm{S}{}^*E_\mathrm{R}}(\mathbf{k}+\mathbf{k}^\mathrm{i}+\mathbf{k}_\mathrm{DG})$ was obtained (Supplementary Figs. 3b, and 3f). Then we got angular spectrum of complex field map in rotating frame $\widetilde{E_\mathrm{S}E_\mathrm{R}{}^*}(\mathbf{k}-\mathbf{k}^\mathrm{i})$ (Supplementary Figs. 3c, and 3g) by shifting the frequency of $\mathbf{k}_\mathrm{DG}$ and

filtering the DC frequency. By taking the inverse Fourier transform of $\widetilde{E_S E_R^*}(\mathbf{k}-\mathbf{k}^{\mathrm{i}})$, complex field map $E_{\mathrm{GM}}(\mathbf{r}_{\mathrm{o}};\mathbf{k}^{\mathrm{i}},\tau_0)$ was obtained (Supplementary Fig. 3d, which is then converted to the map in the laboratory frame for the application of aberration correction algorithm.

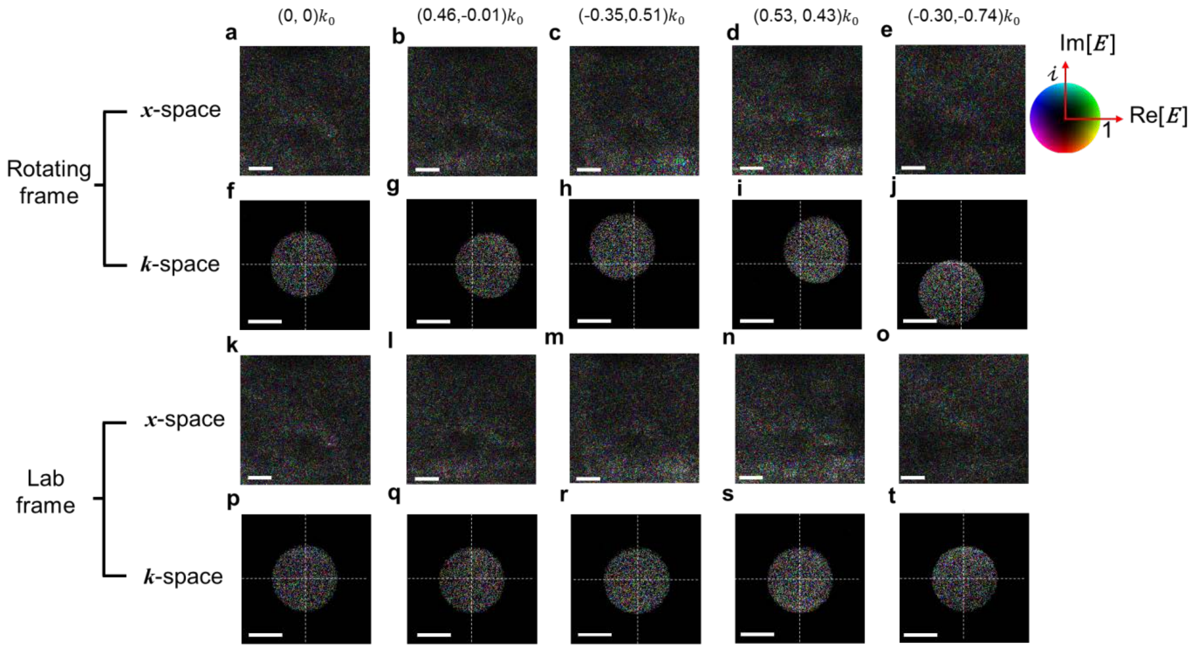

Supplementary Figure 4. Coordinate transformation from rotating reference frame to the laboratory frame. a-e, The complex field maps of the zebrafish for the representative incident wavevectors in the rotating reference frame. **f-j,** The angular spectra of the complex field maps in (a)-(e). **k-o,** The complex field maps for the incident wavevectors corresponding to (**a-e**) in the laboratory frame. **p-t,** The angular spectra of the complex field maps in (**k-o**). Scale bar, 20 μm (**a-e, k-o**), 0.8 k_0NA (**f-j, p-t**). The saturation and color of the color bar indicates the amplitude and phase of the complex field, respectively.

In Supplementary Figs. 4a-e, the complex field maps $E_{\mathrm{GM}}(\mathbf{r}\,;\mathbf{k}^{\mathrm{i}},\tau_0)$ of the zebrafish are shown for the representative incident wavevectors of $\mathbf{k}^{\mathrm{i}}/k_0 = (0,0)$, $(0.46,-0.01)$, $(-0.35,0.51)$, $(-0.53,0.43)$, and $(-0.30,-0.74)$, respectively. All the single-shot images were highly distorted because the specimen had induced strong scattering and aberration. Supplementary Figures 4f-j show the angular spectra of the complex field maps in Supplementary Figs. 4a-e, respectively. The radius of each spectrum corresponds to $0.8k_0$, which is given by the NA of the objective lens. Note that the center of the spectrum is shifted away from the origin as the magnitude of $\mathbf{k}^{\mathrm{i}}$ is increased. This is

because $E_{\mathrm{GM}}(\mathbf{r};\mathbf{k}^{\mathrm{i}},\tau_0)$ contains the reference wave $\left(E_{\mathrm{R}}^{0}\exp[-i\mathbf{k}^{\mathrm{i}}\cdot\mathbf{r}_{\mathrm{o}}]\right)^{*}$. For the application of aberration correction algorithm, we need to extract $E_{\mathrm{S}}(\mathbf{r};\mathbf{k}^{\mathrm{i}},\tau_0)$, which is the complex field map in the laboratory frame. This is done by precisely measuring $\mathbf{k}^{\mathrm{i}}$ from the center of each spectrum in Supplementary Figs.4f-j and shifting each spectrum by $\mathbf{k}^{\mathrm{i}}$ as shown in Supplementary Figs. 4p-t, which is equivalent to normalizing out $\left(E_{\mathrm{R}}^{0}\exp[-i\mathbf{k}^{\mathrm{i}}\cdot\mathbf{r}_{\mathrm{o}}]\right)^{*}$ in $E_{\mathrm{GM}}(\mathbf{r}_{\mathrm{o}};\mathbf{k}^{\mathrm{i}},\tau_0)$. Supplementary Figures 4k-o are the complex field amplitude maps, $E_{\mathrm{S}}(\mathbf{r};\mathbf{k}^{\mathrm{i}},\tau_0)$, in the laboratory frame obtained from the 2D inverse Fourier transform of Supplementary Figs. 4p-t, respectively.

Supplementary Note 4: Construction of the time-gated reflection matrix, $E_S(\mathbf{r}_o; \mathbf{r}_i, \tau_0)$

From a set of $E_S(\mathbf{r}\,; \mathbf{k}^i, \tau_0)$ taken for N_{in} different $\mathbf{k}^i$'s shown in Supplementary Figs. 4k-o, we constructed a time-gated reflection matrix R. Each image was converted to the column vector and appended to the preceding column (Supplementary Fig. 5a). We separately measured the input fields $E_i(\mathbf{r}_o; \mathbf{k}^i, \tau_0)(E_R^0 \exp[-i\mathbf{k}^i \cdot \mathbf{r}_o])^*$ by placing a mirror at the sample plane and converted them to $E_i(\mathbf{r}_o; \mathbf{k}^i, \tau_0)$ after taking away $(E_R^0 \exp[-i\mathbf{k}^i \cdot \mathbf{r}_o])^*$. Since these images were taken for a mirror, $E_i(\mathbf{r}_o; \mathbf{k}^i, \tau_0) = E_i(\mathbf{r}_i; \mathbf{k}^i, \tau_0)$. With these input fields, we constructed a time-gated input matrix P (Supplementary Fig. 5b) in the same way as to construct R. From the product of $R \cdot P^{-1}$, we obtained a time-resolved reflection matrix in position basis $E_S(\mathbf{r}_o; \mathbf{r}_i, \tau_0)$ (Supplementary Fig. 5c), where $\mathbf{r}_i$ is conjugate to $\mathbf{k}^i$.

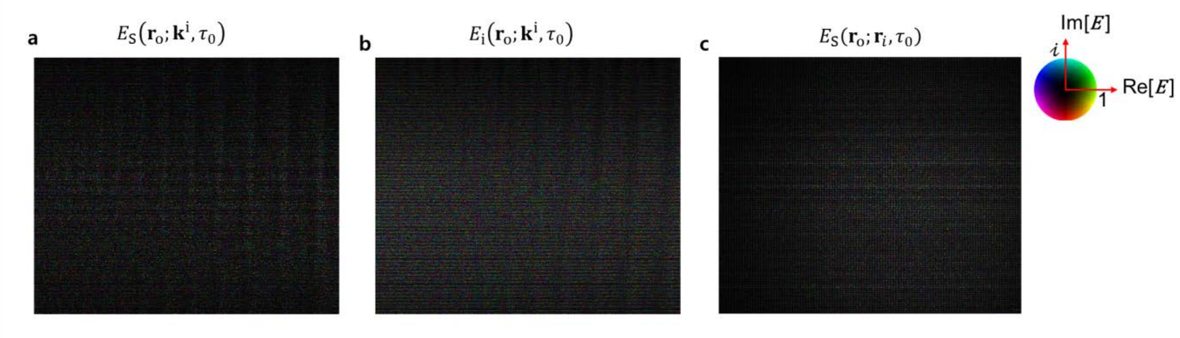

Supplementary Figure 5. Construction of a time-gated reflection matrix, $E_S(\mathbf{r}_o; \mathbf{r}_i, \tau_0)$. a, The time-gated output matrix for input angular basis. **b,** The time-gated input matrix for input angular basis. **c,** The time-gated reflection matrix for position basis. The saturation and color of the color bar indicates the amplitude and phase of the complex field, respectively.

Supplementary Note 5: The application of AO algorithm to each submatrix, $\boldsymbol{E}_{\mathbf{sub}}(\mathbf{r}_{\mathrm{o}};\mathbf{r}_{\mathrm{i}},\tau_0)$

To correct spatially varying aberrations, we decomposed the whole matrix into the sub-matrix $E_{\mathrm{sub}}(\mathbf{r}_{\mathrm{o}};\mathbf{r}_{\mathrm{i}},\tau_0)$ (Fig. 2e) whose size was set by the isoplanatic patch. Here we describe the detailed procedure to apply aberration correction algorithm to $E_{\mathrm{sub}}(\mathbf{r}_{\mathrm{o}};\mathbf{r}_{\mathrm{i}},\tau_0)$ for acquiring $E_{\mathrm{sub}}^{\mathrm{cor}}(\mathbf{r}_{\mathrm{o}};\mathbf{r}_{\mathrm{i}},\tau_0)$ (Fig. 2f). First, we started with the sub-matrix $E_{\mathrm{sub}}(\mathbf{r}_{\mathrm{o}};\mathbf{r}_{\mathrm{i}},\tau_0)$ (Supplementary Fig. 6e) represented in position basis. Because of the strong aberration induced by the zebrafish, $E_{\mathrm{sub}}(\mathbf{r}_{\mathrm{o}};\mathbf{r}_{\mathrm{i}},\tau_0)$ has broad off-diagonal components. The initial PSF (Supplementary Fig. 6j) was significantly distorted. By taking the Fourier transform of $E_{\mathrm{sub}}(\mathbf{r}_{\mathrm{o}};\mathbf{r}_{\mathrm{i}},\tau_0)$, the sub-matrix represented in angular basis, $E_{\mathrm{sub}}(\mathbf{k}^{\mathrm{o}};\mathbf{k}^{\mathrm{i}},\tau_0)$, was obtained (the square matrix in Supplementary Fig. 6a). Then, we multiplied angle-dependent phase shift (Supplementary Fig. 6a, top) to each column of $E_{\mathrm{sub}}(\mathbf{k}^{\mathrm{o}};\mathbf{k}^{\mathrm{i}},\tau_0)$, which maximizes the total intensity of the reconstructed image. This angle-dependent phase shift corresponds to the input aberration of the 1st iteration at pupil plane. Next, we multiplied angle-dependent phase shift (Supplementary Fig. 6b, left) to each row of the sub-matrix corrected by the previous step (the square matrix in Supplementary Fig. 6b) in such a way to maximize the total intensity of the reconstructed image. This angle-dependent phase shift corresponds to the output aberration of 1st iteration at pupil plane. Throughout this closed loop, the sub-matrix corrected by one iteration, $E_{\mathrm{sub}}^{\mathrm{cor}(1)}(\mathbf{k}^{\mathrm{o}};\mathbf{k}^{\mathrm{i}},\tau_0)$, was retrieved. By taking the inverse Fourier transform of this corrected matrix, the position basis representation, $E_{\mathrm{sub}}^{\mathrm{cor}(1)}(\mathbf{r}_{\mathrm{o}};\mathbf{r}_{\mathrm{i}},\tau_0)$, was reconstructed (Supplementary Fig. 6f), which shows the enhanced diagonal components and reduced off-diagonal components. The PSF corrected by this first round of iteration (Supplementary Fig. 6k) became sharper than before and its peak intensity was greatly increased.

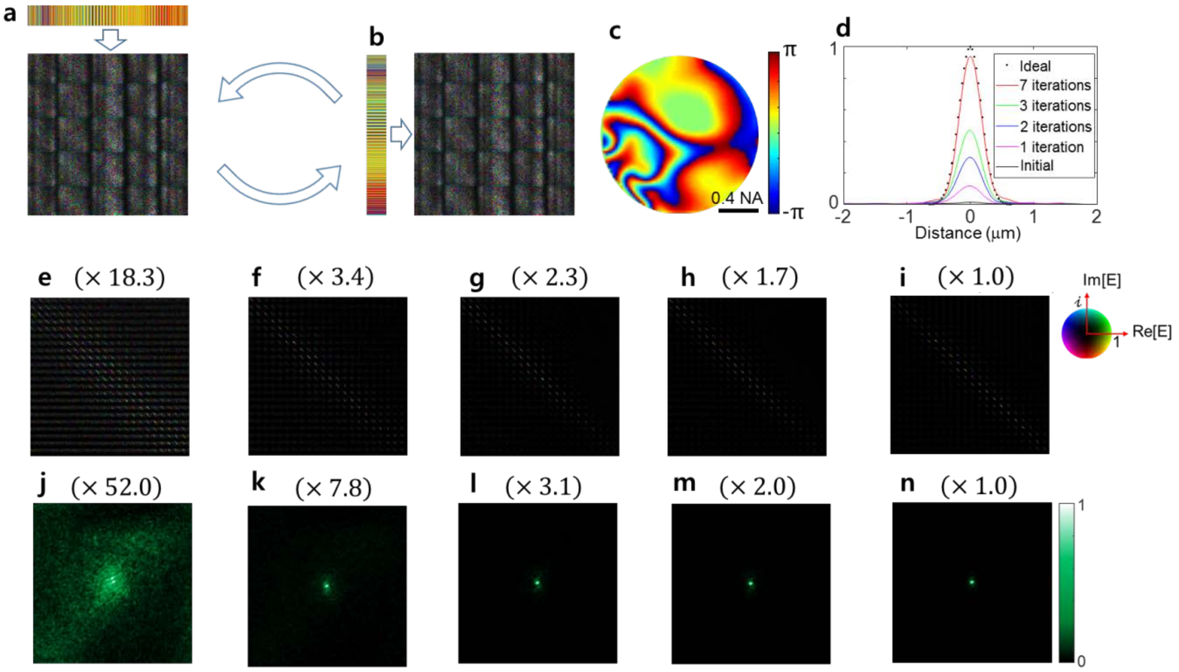

Supplementary Figure 6. Application of AO algorithm for the single submatrix, $E_{\mathrm{sub}}(\mathrm{r_o}; \mathrm{r_i}, \tau_0)$. **a, b,** Determination of the angle-dependent phase retardation in the sub-matrix represented in the wavevector basis at the input and output paths. **c,** Sample-induced aberration determined by the correction algorithm. **d,** The line profiles of the PSF before and after the aberration correction. **e- i,** The sub-matrices represented in the position basis, and initial, and after 2, 3, 7 round of iterations, respectively. **e- i,** The PSFs corresponding to the sub-matrices of **e- i**, respectively. Color bar, intensity normalized by the maximum value.

Supplementary Figures 6g-i show the sub-matrices $E_{\mathrm{sub}}^{\mathrm{cor}(2)}(\mathbf{r}_\mathrm{o}; \mathbf{r}_\mathrm{i}, \tau_0)$, $E_{\mathrm{sub}}^{\mathrm{cor}(3)}(\mathbf{r}_\mathrm{o}; \mathbf{r}_\mathrm{i}, \tau_0)$ and $E_{\mathrm{sub}}^{\mathrm{cor}(7)}(\mathbf{r}_\mathrm{o}; \mathbf{r}_\mathrm{i}, \tau_0)$ after 2, 3, and 7 round of iterations, respectively, and Supplementary Figs. 6l-n show the respective PSFs. With the increase of iteration number, the diagonal components of the matrix became gradually distinct and the PSF eventually converged to a clear spot. Supplementary Figure 6d shows the line profiles of the PSF before and after the aberration correction. The line profile of the PSF after 7 rounds of iterations agrees well with the theoretical prediction (black dots). The absolute value of Strehl ratio was 0.94, confirming that the PSF reaches almost the diffraction-limit spot. Supplementary Figure 6c shows the final aberration map obtained by accumulating the angle-dependent phase shift during 7 iterations. To compare the absolute scale of the sub-matrices and PSFs during the iterations, the image amplitude and intensity were multiplied by the factors shown above

the figures.

The computation time depends on the field of view and the number of segments. When a personal computer with a CPU clock speed of 3.6 GHz was used, it took about 20~30 minutes for reconstructing the coherent volume of $110 \times 110 \times 10\ \mu\text{m}^3$. The iterative aberration correction for a single segment of $22 \times 22\ \mu\text{m}^2$ took about an order of ten seconds. It took about tens of minutes to calculate *en face* image of the full field of $110 \times 110\ \mu\text{m}^2$, which is composed of 6×6 segments. In addition, it took about ten minutes to computationally propagate the acquired *en face* image to 10 different depths within the coherent volume of $110 \times 110 \times 10\ \mu\text{m}^3$. Total computation time for a whole central nerve system encompassing the hindbrain of the zebrafish shown in Fig. 3, which consists of 120 coherent volumes or 43,200 segments, was around 50 hours. However, there are many rooms to optimize the computation since the current algorithm is sequentially dealing with each segment. The total computation time could be extremely reduced by parallel processing and GPU computing for FFT with sufficient computing power.

Supplementary Note 6: Maximum achievable image acquisition speed depending on the degree of aberration

The effective image acquisition speed excluding the image processing step in AO-SASM is mainly determined by the camera frame rate and the number of raw images N_{in} given by the number of $\mathbf{k}^{\mathrm{i}}$'s required for the aberration correction. Camera frame rate is given by the camera hardware and the region of interest. The camera used in our experiment has the frame rate of 450 Hz for 400×400 pixels, which corresponds to the view field of $22 \times 22\ \mu\mathrm{m}^2$. In the case of N_{in}, it depends on the degree of aberration and multiple scattering noise, but here we mainly verified its dependence on the degree of aberration. Note that each raw image contains the aberration information for all the output modes, which is the same as that of input modes in the epi-detection geometry. If the target object is a point particle, single raw image should be good enough to map out the aberration. In the case of extended objects, multiple images taken for various $\mathbf{k}^{\mathrm{i}}$'s are required to determine the wavefront in the given isoplanatic patch. Theoretically, the best performance for aberration correction is expected when N_{in} is equal to the number of free modes N_{max} given by the number of diffraction-limit spot in the view field. N_{max} is 2,285 in the case of $22 \times 22\ \mu\mathrm{m}^2$ view field and the diffraction-limit resolution of 480 nm. However, the aberration correction can reach the diffraction-limit resolution with much smaller number of angular scans N_{in} than N_{max} even for the strong aberration that attenuates Strehl ratio by 100 or more. The aberration correction process introduced in Supplementary Note 5 is perfectly compatible with the case when N_{in} is much smaller than N_{max}.

We experimentally verified the minimum required N_{in} for achieving the diffraction-limit spatial resolution depending on the degree of aberration. Supplementary Figure 7 shows the imaging of a Siemens star structure placed below highly aberrating medium made of Low Density Polyethylene (LDPE). Supplementary Figures 7a-e show the results without the aberrating layer. Therefore, diffraction-limit resolution was obtained for confocal reflectance (Supplementary Fig. 7a), time-gated reflection matrix before the aberration correction (Supplementary Fig. 7b). By the aberration correction (Supplementary Fig. 7c), we verified that the system aberration was negligible from the

aberration map (Supplementary Fig. 7d) and its decomposition into Zernike polynomials (Supplementary Fig. 7e). N_{in} used for the time-gated reflection matrix was 50. After placing one layer of LDPE, we acquired the same set of data and observed that confocal reflectance (Supplementary Fig. 7f) and image before the aberration correction (Supplementary Fig. 7g) show degraded resolving power. On the other hand, the fine patterns at the diffraction-limit spatial resolution were recovered by applying aberration correction algorithm (Supplementary Fig. 7h) using just $N_{\mathrm{in}} = 100$ raw images. The identified aberration map (Supplementary Fig. 7i) and its Zernike polynomial decomposition (Supplementary Fig. 7j) indicates that high-order modes of the aberration were introduced by the medium. By the analysis of the PSF as described in Supplementary Note 5, the estimated enhancement of Strehl ratio was 23. We added one more layer of LDPE and applied aberration correction algorithm to recover the diffraction-limit spatial resolution (Supplementary Fig. 7m). The aberration map (Supplementary Fig. 7n) was much more complex than that for a single LDPE layer. And the estimated Strehl ratio enhancement was 162. Despite the complexity of aberration, sparse angular scanning of $N_{\mathrm{in}} = 150$ offered the optimal resolution. With the camera frame rate of 450 Hz, data acquisition time for Supplementary Figs. 7h and 7m was only 0.22 and 0.33 seconds, respectively, to complete all the image acquisition even with this significant aberration. And these correspond to the aberration correction speed of 10,300 modes/s, and 6,900 modes/s, respectively.

Reducing the number of incidence angles, N_{in}, affects to the achievable imaging depth. The coherent accumulation of multiple angular images enhances single scattering signal to multiple scattering noise ratio by N_{in} times (Ref. 31 in the main text). Therefore, image reconstruction by a smaller number of angular images becomes more susceptible to the multiple scattering noise. For example, the system SNR reaches 74 dB when $N_{\mathrm{in}} = 3{,}000$ while it is only about 39.4 dB when $N_{\mathrm{in}} = 1$. This is why conventional software-based AO starting from single complex-field map (Ref. 30 in the main text) is prone to the multiple scattering noise. A good strategy to improve data acquisition speed is to use smaller number of illumination angles at a shallower depth and to increase N_{in} with the increase of depth.

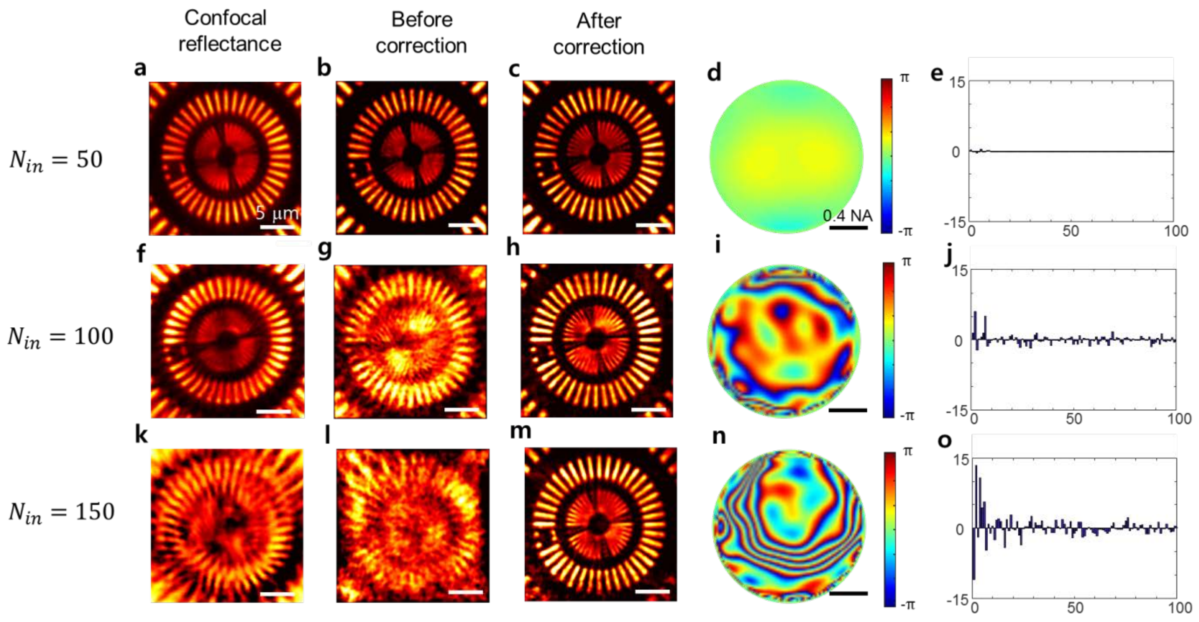

Supplementary Figure 7. Recovery of the diffraction-limit spatial resolution with minimal number of raw images N_{in} for the aberration correction. a-o, Imaging of a Siemens star without aberrating medium (**a-e**), under the mild (**f-j**), and strong (**k-o**) aberrating medium, respectively. The images acquired by confocal reflectance (**a, f, k**), time-gated reflection matrix before the aberration correction (**b, g, l**), and after the aberration correction (**c, h, m**), respectively. **d, i, n,** The aberration map. **e, j, o,** decomposition of the aberration into Zernike polynomials. Color bar, phase retardation in radians.

Further, we investigated the imaging performance to verify the benefits of our algorithm by separately correction of input and output aberrations in comparison with well-known computational correction algorithm. In the OCT and OCM imaging, focused illumination and pinhole detection form a confocal gating. Previously, in various studies reported techniques of computational adaptive optics such that the aberration correction map based on Zernike polynomials was applied to the Fourier transformed angular spectrum map of the OCM complex-field map in such a way to maximize the image sharpness metric defined by the summation of intensity squared in the image. This algorithm has a major limitation in the case of high-resolution imaging. The Fourier transformed image contains aberrations for both illumination and collection beam paths. Therefore, applying correction to this image cannot deal with the aberration of illumination separately from that in the collection beam path. This may not be a significant issue in the case of low numerical-aperture (NA) imaging, but in high NA imaging the aberration in the illumination beam path causes significant broadening of the

illumination PSF.

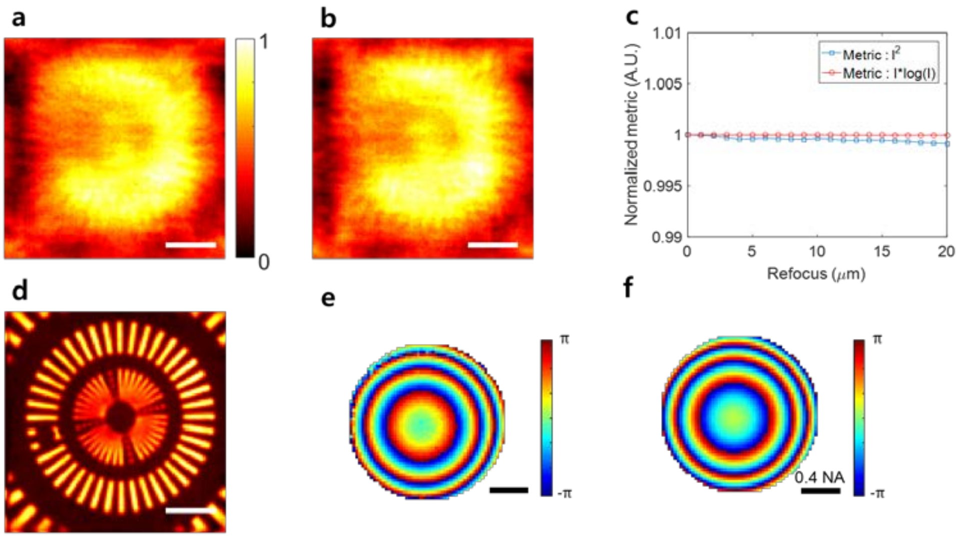

Supplementary Figure 8. Comparison of aberration correction performance with software-based approach using optical coherence microscopy for high-NA imaging. a, Amplitude map numerically defocused by -10 μm for OCM image of a Siemens star pattern. Scale bar: 5 μm. **b,** Amplitude map after applying 10 μm refocusing to the angular spectrum of **a**. **c,** Variation of image metric with the scanning of the coefficient of the Zernike polynomial corresponding to defocusing. **d,** AO-SASM image for the same Siemens star pattern in **a** after applying our algorithm. **e** and **f,** aberration maps for input (**e**) and output (**f**) paths. Color bars: phase in radians.

To elucidate this point, we eliminated both input and output aberrations in the measured time-gated reflection matrix. We then added defocus aberration by -10 μm to both input and output paths. Supplementary Figure 8a shows the resulting angular scanned coherently accumulated image of a resolution target before the application of aberration correction, which is equivalent to conventional OCM image. We then scanned the coefficient of the fourth-order Zernike polynomial applied to the Fourier transformed complex-field map of the OCM image. Supplementary Figure 8c shows the image metric with the scan of the computational refocusing distance using the same method as the reference papers. Two representative image metrics, summation of intensity square and Shannon entropy, were estimated, but there were no perceivable peaks at 10 μm. And the computational refocusing back to 10 μm didn't show a clear image (Supplementary Fig. 8b). This is because the aberration in the illumination path was not dealt with in this reference work. On the other hand, AO-

SASM found the aberration maps for both illumination and collection paths (Supplementary Figs. 8e and f), which correspond to 10 μm defocus, and showed clear object image (Supplementary Fig. 8d). The main difference of AO-SASM from conventional pinhole-gated OCM is that AO-SASM collects signals at both the point of illumination and the surrounding area. This ultimately enables us to deal with the input and output aberrations separately, thereby enabling high-order aberration correction for high-NA imaging.

Supplementary Note 7: Maximum achievable image acquisition speed for the biological specimens

We verified that the minimum required N_{in} for achieving diffraction-limit spatial resolution can be much smaller than N_{max} in case of biological specimens as well. Supplementary Figure 9 shows in vivo imaging of the hindbrain of a 12 dpf zebrafish. Supplementary Figure 9a shows confocal fluorescence MIP image for the depth range between 80 μm and 290 μm. We performed AO-SASM imaging in the area marked with white and red boxes in Supplementary Fig. 9a. The field of view was $22 \times 22\ \mu\mathrm{m}^2$ and the camera frame rate was 400 Hz. Supplementary Figures 9b-e show confocal reflectance image, AO-SASM images before and after the aberration correction, and aberration map, respectively, for the white box in Supplementary Fig. 9a. Likewise, Supplementary Figs. 9f-i show the imaging results for the red box in Supplementary Fig. 9a. In both cases, AO-SASM with aberration correction show clear visualization of myelin processes. From the identified aberration map (Supplementary Figs. 9e and 9i), the estimated enhancement of Strehl ratio was 115 and 27, respectively. And the required number of raw images was $N_{\mathrm{in}} = 200$, for which it took 0.5 seconds to complete the image acquisition. This correspond to the aberration correction speed of 4,600 modes/s.

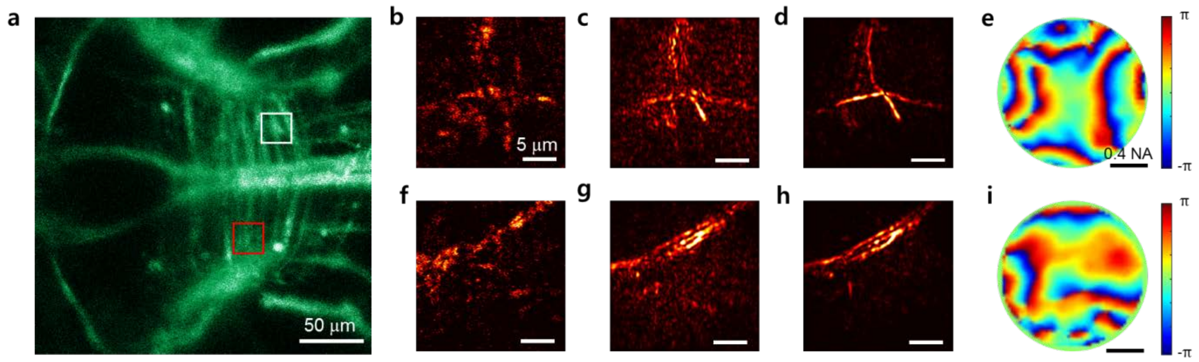

Supplementary Figure 9. High-speed imaging of a living zebrafish. a, The confocal fluorescence MIP image for the depth range between 80 μm and 290 μm. **b-h,** The images acquired by confocal reflectance (**b, f**), time-gated reflection matrix before the aberration correction (**c, g**), and the aberration correction (**d, h**). **e, i,** The aberration map. Color bar, phase retardation in radians.

Supplementary Note 8: Additional depth-dependent images for the zebrafish shown in Fig. 2

In Fig. 2 of the main text, we visualized the neuropil contiguous to the ear at the depth of 160 μm in the living zebrafish. For the same zebrafish, we also took time-gated reflection matrices at the depths of 140 μm and 180 μm, and reconstructed images at the respective depths. To verify that the fine structures identified by AO-SASM are myelinated axons, we took confocal fluorescence images of the transgenic zebrafish (Tg(*claudinK:gal4;uas:mgfp*)) with the excitation wavelength of 473 nm. In addition, confocal reflectance images were taken with the source wavelength of 473 nm to see the effect of aberration for the conventional imaging modality. It is noteworthy that the structures of myelinated axons were clearly distinct at each depth (Supplementary Figs. 10b, g, l), and the aberration maps (Supplementary Figs. 10e, j, o) were also highly depth-dependent. Confocal fluorescence images (Supplementary Figs. 10d, i, n) showed similar structures to AO-SASM images (Supplementary Figs. 10b, g, l), but many of the fine myelin processes were not clearly resolved due to the aberration. Confocal reflectance images (Supplementary Figs. 10c, h, m) were rather similar with those AO-SASM images before aberration correction (Supplementary Figs. 10a, f, k) with more background noise due to the absence of temporal gating.

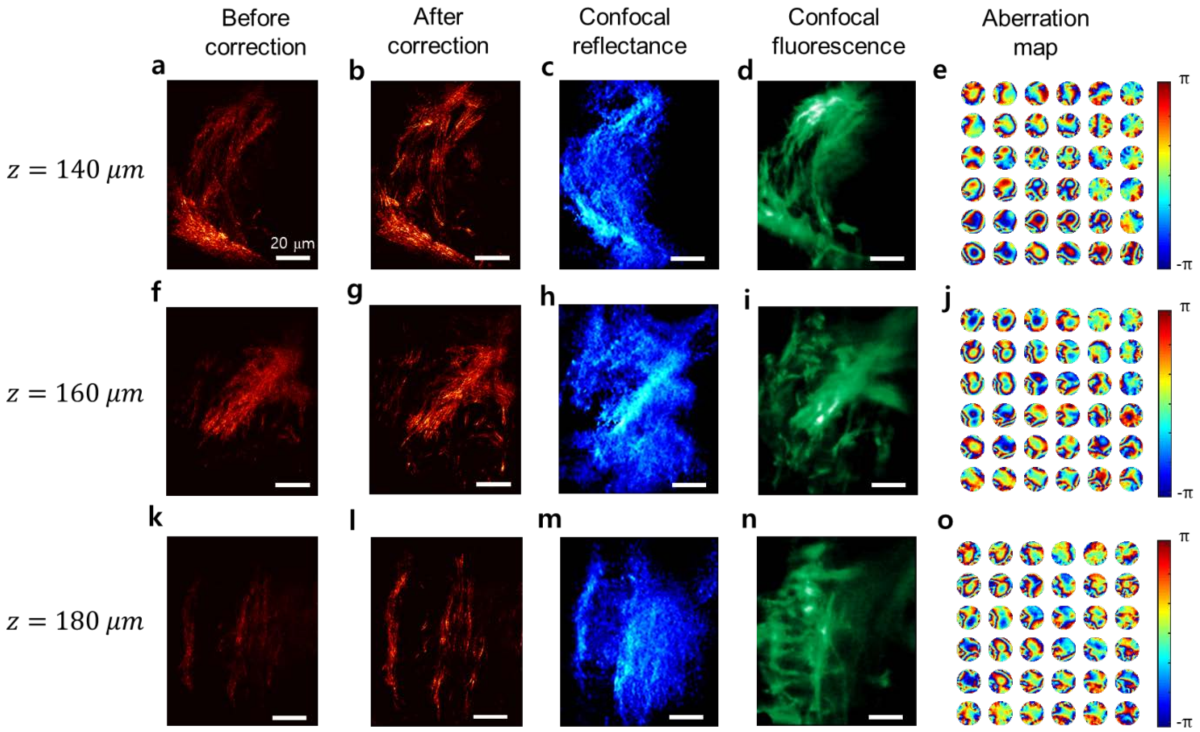

Supplementary Figure 10. Comparison of AO-SASM with confocal reflectance/fluorescence microscopy. a-o, In vivo imaging of a larval zebrafish at the depths of 140 μm (**a-e**), 160 μm (**f-j**), 180 μm (**k-o**), respectively. The images acquired by time-gated reflection matrix before the aberration correction (**a, f, k**), after the aberration correction (**b, g, l**), and the confocal reflectance (**c, h, m**), and the confocal fluorescence (**d, i, n**), respectively. **e, j, o,** The aberration map. Color bar, phase retardation in radians.

Supplementary Note 9: Approximate estimation of the scattering mean free path for the zebrafish shown in Fig. 3

Scattering mean free path of biological tissues is a difficult quantity to measure, especially in the case of in vivo imaging. To precisely assess the scattering mean free path, we need to measure ballistic photons by varying the thickness of the same type of scattering medium. In the case of zebrafish imaging, scattering property varies from depth to depth because the internal structures change with depth as shown in Figs. 3a-e in the manuscript. One approximate way for estimation is to measure the average intensity of confocal reflectance imaging, which is a measure of ballistic photons, as a function of depth. To do that, we analyzed here a 10 dpf zebrafish data (Supplementary Fig. 11a) taken at the wavelength of 633 nm. Supplementary Figure 11b shows the average intensity as a function of depth for the white dotted rectangular area in Supplementary Fig. 11a. Supplementary Figure 11c was acquired from the blue dotted-square area in Supplementary Fig. 9a. The intensity profiles cannot be fit to a single exponential curve (red curves) partly because confocal reflectance imaging picks up multiple scattering noise at the depths deeper than 50-100 μm and partly because scattering properties varied with depth.

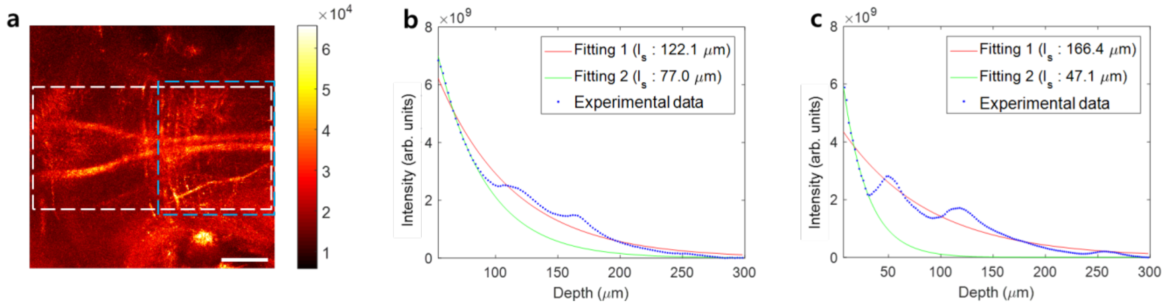

Supplementary Figure 11. Scattering property of a 10-dpf larval zebrafish. a, Dorsal view of confocal image encompassing the hindbrain. **b** and **c,** Intensity profiles along the depth in the white dashed rectangle (**b**) and blue dashed square (**c**), respectively.

Therefore, we performed exponential curve fittings up to a shallow depth (green curves). The approximate scattering mean free paths were around 50 to 100 μm. Considering the double-pass detection geometry, this corresponds to the attenuation of single scattering intensity by a factor of 10^2-10^4 times at the depth of 200 μm in the case of normal-illumination complex-field map. The sample-induced aberrations attenuate single scattering intensity by an additional factor of 10~100 in the

image formation step. The constructive superposition of multiple angular waves is compromised due to the angle-dependent phase retardations induced by the sample. This explains why myelinated axons were invisible in the individual complex-field maps.

Supplementary Note 10: The benefit of dealing with aberrations for all the angular modes

We analyzed the necessity of correcting all the angular modes in the view field using the data shown in Supplementary Figs. 7k-o. The first column in Supplementary Fig. 12a show the aberration map, and the reconstructed AO-SASM image. We decomposed the aberration maps into Zernike polynomials. Supplementary Figure 12b shows amplitudes of various orders of Zernike polynomials constituting the aberration map of AO-SASM. One can notice that there are non-negligible contributions even for the 1000th order of the polynomials. We then reconstructed aberration-corrected image after using only the first 50 orders of polynomials of Supplementary Fig. 12b.

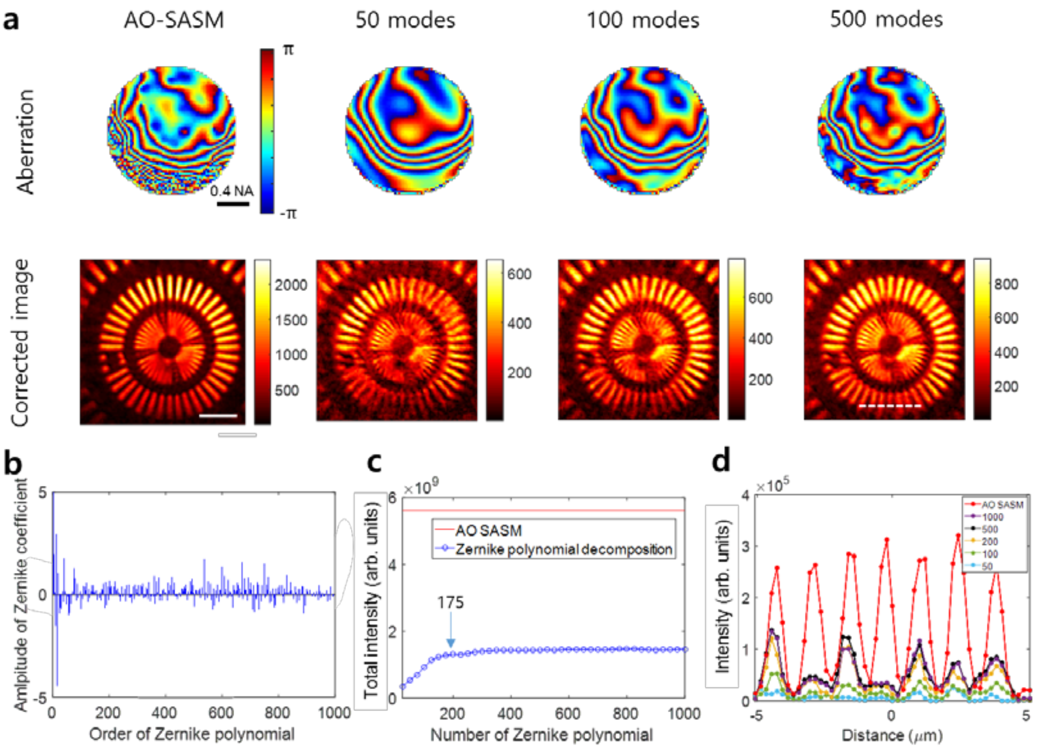

Supplementary Figure 12. Image reconstruction with finite orders of Zernike polynomials. a, The first column shows aberration map and reconstructed image in the case of AO-SASM. The second, third and fourth columns correspond to the cases of using 50, 100, and 500 Zernike polynomials. **b,** Decomposition of the aberration identified by AO-SASM into various orders of Zernike polynomials. **c,** Total intensity of reconstructed images in **a** as a function of the number of orders of polynomials. Red line indicates the case of AO-SASM. **d,** Intensity profiles along the dashed line shown in **a** depending on the number of orders of polynomials. Red dots were derived from AO-SASM image.

The aberration maps and reconstructed image are shown at the second column in Supplementary Fig. 12a. The aberration was so severe that structures remained blurred even with the use of 50 orders. And

the signal level of the reconstructed image with 50 Zernike modes is much smaller than that of AO-SASM image since the intensity is proportional to the Strehl ratio. The third and fourth columns of Supplementary Fig. 12a show the results with the use of 100 and 500 orders, respectively. We can observe the gradual increase of image quality, but the sharpness, intensity, and signal to background ratio are still below the level shown by AO-SASM. Specifically, Supplementary Fig. 12c shows the total intensity of the reconstructed with the increase of the number of Zernike polynomial orders. The total intensity increased up to the use of 175 orders and reaches the saturation level, which is far lower than the total intensity of AO-SASM. Note that conventional software-based AOs seldom correct aberration even up to 100 orders, where the aberration correction is hardly ideal. This tells us that correction of aberrations in the angular basis is critical and advantageous in dealing with the severe aberrations. This is partly because Zernike polynomials cannot recover abrupt variations of aberration in the pupil and partly because angular basis is orthogonal and complete in the electric field. In Supplementary Fig. 12d, we also plotted the intensity profiles along the dashed line shown in Supplementary Fig. 12a as a function of the number of polynomial orders. We can notice that only AO-SASM can faithfully recover the fine details of structures. This confirms that the use of full angular modes is critical to obtain spatial resolution close to ideal spatial resolution especially when aberration in the pupil plane varies pixel-by-pixel level.

Supplementary Note 11: The changes of single- and multiple-scattered waves in their intensity with respect to the iteration number

Our AO-SASM algorithm corrects aberration and suppresses speckle noise at the same time. We coherently superpose multiple images taken at different illumination angles. When there exist aberrations, this coherent addition is not fully constructive for the single scattering signal, and the intensity of superposed image is reduced as a consequence. Therefore, we identify the angle-dependent phase correction factors to maximize the total intensity of the reconstructed image. Note that this optimization of total intensity preferably affects to the single scattering. If multiple scattering produces random speckles, their total intensity in the view field doesn't vary much by the addition of the aberration-correction phase factors. For this reason, the algorithm used in AO-SASM works even in the presence of strong multiple scattering noise.

To support the working mechanism of our algorithm, we performed a new data analysis for the image taken from 12 dpf zebrafish (Supplementary Fig. 13). Supplementary Figure 13a is a time-gated reflection image for a normal illumination, and Supplementary Fig. 13b is the coherently superposed image of 800 angular images with no aberration correction, which corresponds to conventional OCM image. Single-angle time-gated image (Supplementary Fig. 13a) was mostly dominated by multiple scattering noise, and it is hard to conceive whether there is any structure or not. Coherently superposed image is better than single-angle image as the existence of myelinated axons can be conjectured. However, the broadening of PSF makes single scattering still smaller than multiple scattering noise. Supplementary Figures 13c and d show the images after forward and backward optimization processes, respectively, and Supplementary Fig. 11e was acquired after 5 iterations. We could observe that the ratio of single scattering signal to multiple scattering noise was greatly improved with the increase of iteration number. For the quantitative analysis, we calculated average multiple scattering intensity per pixel over the area indicated by the square box in Supplementary Fig. 13b where there were no myelinated axons and monitored its variation with respect to the iteration number (black dots in Supplementary Figs. 13f and g). And we computed the average intensity per pixel along the lines where myelinated axons were located. Red dots in Supplementary Fig. 13f were

derived from the area indicated by a white ellipse in Supplementary Fig. 13b, and those in 13g from the yellow ellipse in Supplementary Fig. 13b. In each case, we obtained average single scattering intensity per pixel by computing the difference between the intensity at the myelinated axons and that in the background (blue dots in Supplementary Figs. 13f and g). In accordance with the theory and numerical analysis, we could confirm that the increase of the total intensity by the iterative optimization was mainly due to the increase of single scattering intensity. In Supplementary Fig. 13h, we show the amplitude profile along the white line in Supplementary Fig. 13c, where we could observe that the background noise level stays almost the same, and signal was increased only at the myelinated axons.

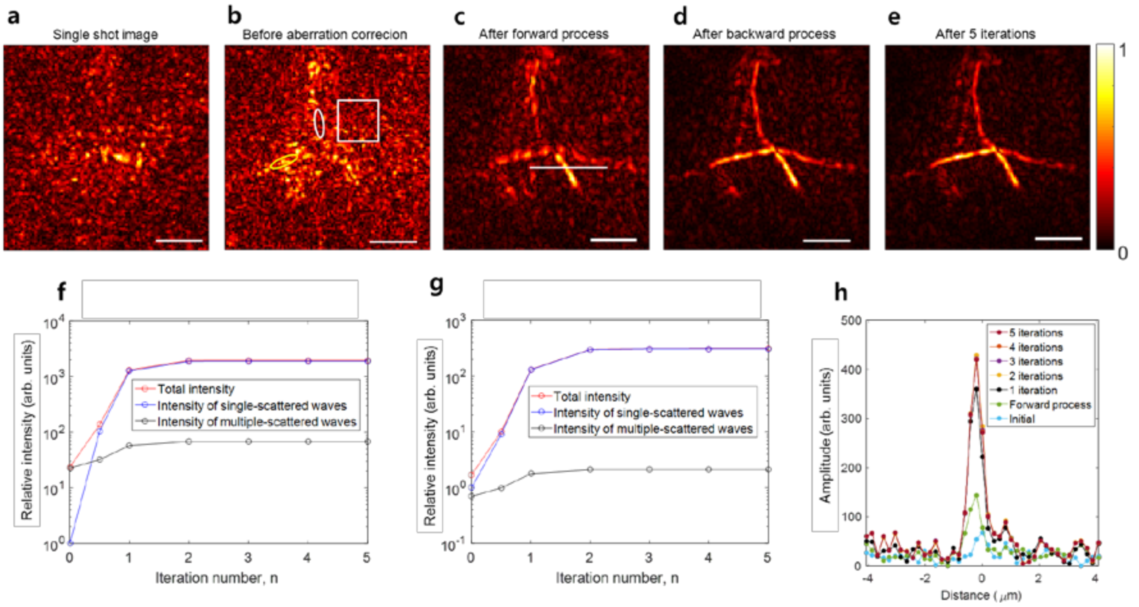

Supplementary Figure 13. Image analysis with the increase of iteration number. a, Amplitude map of a complex-field map acquired for the normal illumination. **b,** Amplitude map after coherent superposition of complex-field maps taken for 800 different illuminations. Aberration correction was not yet applied. **c** and **d,** Amplitude maps after forward and backward optimizations of the first iteration, respectively. **e,** Amplitude map after 5 iterations. **f,** Average intensity per pixel within the white ellipse in **b** (red dots) and that in the square box in **b** (black dots). Their difference is plotted in blue dots, which corresponds to single scattering signal intensity. **g,** Same as **f**, but within the yellow ellipse in **b**. **h,** Amplitude along the white line in **c** with the increase of iteration number. Scale bar in **a**: 5 μm.

Supplementary Note 12: Axial resolution of AO-SASM and 3D imaging within coherence volume

In our experiment, the bandwidth of light source was set 15 nm, which corresponds to the temporal coherence time of 100 fs. This corresponds to the coherence length of 30 μm, which is translated into the depth gating of 15 μm after accounting for the epi-detection geometry. And depth resolution set by the confocal gating was measured to be 2.0 μm (Supplementary Fig. 14a), which is close to the theoretical resolution given by $\frac{0.88\lambda}{(n-\sqrt{n^2-NA^2})}$~2.0 μm. This axial resolution by the confocal gating is much shorter than coherence gating due to the use of 0.8 NA objective lens. The axial intensity profile shown in Supplementary Fig. 14a was measured for a reflecting surface. Its FWHM was 1.7 μm, and the distance from the center to the first minima was measured to be 2.0 μm.

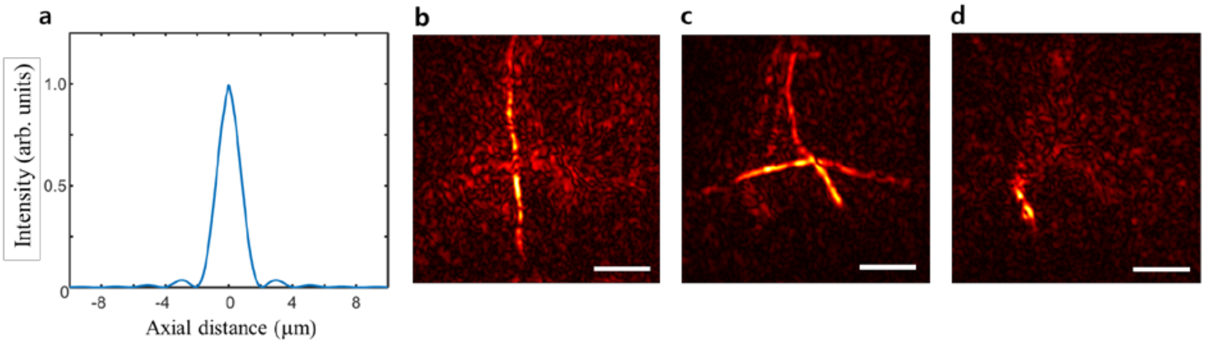

Supplementary Figure 14. Axial resolution of the system and computational refocusing to different depths within coherence volume. a, Intensity profile along the depth measured for a reflecting surface with 800 angular incident waves. **b-d,** Computational refocusing within a single coherent volume. **c**: image at the original focus. **b** and **d**: images after numerical propagation by -5 μm and 5 μm from the original focus. Scale bar, 5 μm.

The bandwidth of 15 nm was chosen for a reasonable temporal gating to reject multiple scattering from other depths and also for a volumetric imaging within the 15 μm-thick coherence volume. AO-SASM acquires wide-field coherent images, which enables 3D imaging within the coherence volume by the axial resolution of the objective lens. In other words, multiple depth images can be acquired over the range of 15 μm by the axial resolution of 2.0 μm. This can be done by computationally propagating each complex-field image taken at an original depth to a desired depth within the

coherence volume. Supplementary Figures 14b-d show an example of computational refocusing to different depths from the data acquired at an initial depth.

For the optimal axial resolution and imaging depth, the bandwidth of laser should be enlarged to 15 nm or larger such that coherence gating is comparable to the confocal gating. However, this is subject to the system dispersion, which is especially difficulty to eliminate for wide-field interferometric imaging with rotating illuminations. And the number of depth images required to cover the sample volume becomes impractically large.

Supplementary Note 13: The SNR of AO-SASM system

We described here how the SNR of AO-SASM is determined. At first, we measured the SNR of single-angle complex-field map. Supplementary Figure 15a shows the intensity of the complex-field map for the normal illumination as a function of the position of the reference mirror. The blue dots were acquired when there was a reflector at the sample plane, and the red dots were obtained when there was no reflector at the sample plane. The signal to background ratio (SBR) defined by the ratio of signal intensity to background intensity was measured to be about 39.7 dB. Signal to noise ratio (SNR), defined by the ratio of signal intensity to the standard deviation of background intensity, was measured to be 39.4 dB.

In AO-SASM, complex-field maps taken for different illumination angles are coherently superposed. Then, both the SBR and SNR increase in proportion to the number of illumination angles, N_{in} (ref. 28). Supplementary Figures 15c and d show the AO-SASM images with and without a reflector at the sample plane in the case of $N_{\mathrm{in}} = 3{,}000$. The SBR in this case was measured to be about 74.0 dB, about 34 dB higher than the single-angle imaging as expected. And SNR was measured to be about 74.0 dB, similar to SBR. The background noise was measured by the standard deviation of the image in Supplementary Fig. 15d. Supplementary Figure 15b shows the SBR and SNR as a function of N_{in}.

The SNR of our system is about 20-30 dB lower than the point-scanning FD-OCT partly because our detection scheme is in the temporal domain. Typically, the dynamic range of the spectral domain approach is about 20 dB higher than the temporal domain approach. Another reason is the effect of specular reflections from various optical elements. Our system is wide-field detection with high numerical aperture, and it is even equipped with the synchronous angular scanning setup. Therefore, it is likely that unwanted reflections can contribute to the noise. In fact, bright spots in Supplementary Fig. 15d are due to the artefact. Considering all these effects, we think that 70 dB SNR is a reasonable level. In particular, with this SNR, we could measure single scattering signal when the intensity of multiple scattering is 70 dB higher.

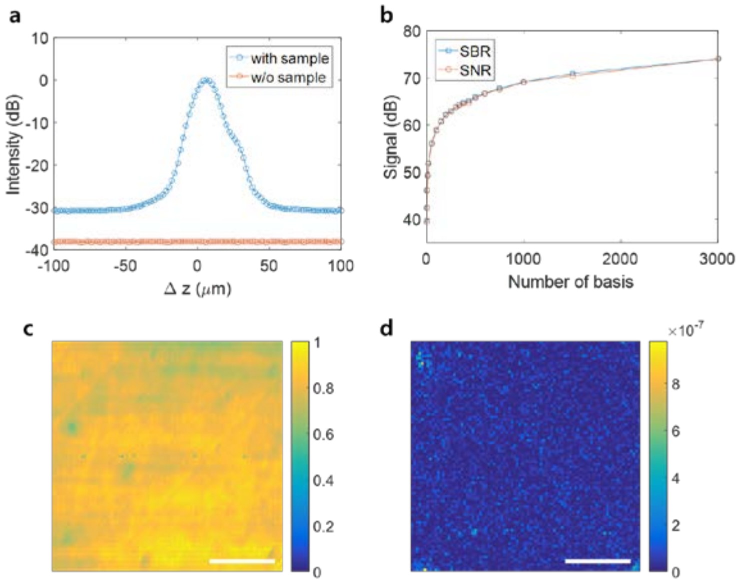

Supplementary Figure 15. Sensitivity and SNR of the system. a, Intensity profile along the depth for normal incident wave. Blue circles, for the reflecting surface red circles, without the reflecting surface at the sample arm. **b,** SBR and SNR vs number of angular basis. **c, d,** Coherently accumulated images for 3,000 incident angular basis for the reflecting surface (c), for w/o the reflecting surface (d). Scale bar, 5 μm. Color bar, normalized intensity.
