# Supplementary material for: Label-free neuroimaging in vivo using synchronous angular scanning microscopy with single-scattering accumulation algorithm
Source: Nat Commun. 2019 Jul 17;10:3152. doi: 10.1038/s41467-019-11040-z (PMC6637127; doi:10.1038/s41467-019-11040-z)
Supplement: Supplementary file 3 — Description of Additional Supplementary Files [file 41467_2019_11040_MOESM3_ESM.pdf]

Description of Additional Supplementary Files

File Name: Supplementary Movie 1
Description: Depth-dependent slice images of the 10-dpf zebrafish shown in Fig. 3a

File Name: Supplementary movie 2
Description: 3D rendered tomography of the 10-dpf zebrafish shown in Fig. 3a
